# Effects of regular exercise on ischemia-modified albumin and total sulfhydryl levels in young women: a cross-sectional study

**DOI:** 10.3389/fphys.2025.1719454

**Published:** 2025-11-20

**Authors:** Ömer Faruk Bilici, Muhammed Zahit Kahraman, Ali Türker, Sinan Seyhan, Mehmet Furkan Sahin, Halit Demir, Görkem Acar, Muhammed Fatih Bilici, Caglar Soylu, Tarkan Söğüt, Abdullah Bakum

**Affiliations:** 1 Institute of Health Sciences, Marmara University, İstanbul, Türkiye; 2 Faculty of Sports Sciences, Bitlis Eren University, Bitlis, Türkiye; 3 Recreation Management, Faculty of Applied Sciences, Karamanoğlu Mehmetbey University, Karaman, Türkiye; 4 Department of Coaching Education, Faculty of Sport Sciences, Manisa Celal Bayar University, Manisa, Türkiye; 5 Coaching Education, Faculty of Sports Sciences, Muş Alparslan University, Muş, Türkiye; 6 Faculty of Science, Van Yüzüncü Yıl University, Van, Türkiye; 7 Department of Sport Science, Institute of Graduate Education, Manisa Celal Bayar University, Manisa, Türkiye; 8 Faculty of Sports Sciences, Muş Alparslan University, Muş, Türkiye; 9 Gulhane Faculty of Physiotherapy and Rehabilitation, University of Health Sciences, Ankara, Türkiye; 10 Department of Coaching Education and Sports, Siirt Universirty, Siirt, Türkiye; 11 Faculty of Sports Sciences, Selcuk University, Konya, Türkiye

**Keywords:** ischemia-modified albümin, sulfhydryl groups, oxidative stress, redox homeostasis, exercise physiology, women

## Abstract

**Background:**

Regular physical activity provides systemic health benefits, including improvements in redox homeostasis and antioxidant defense. Ischemia-modified albumin (IMA) and total sulfhydryl groups (–SH) serve as sensitive biomarkers of oxidative protein modification and thiol-dependent antioxidant capacity. However, evidence regarding their relationship in young women who participate in structured exercise remains limited. This study aimed to investigate associations between regular exercise and serum IMA and –SH concentrations in healthy young women to better understand potential exercise-related redox differences and sex-specific physiological profiles.

**Methods:**

This cross-sectional study included 30 healthy women aged 18–25 years, recruited from university campuses and local fitness facilities. Participants were assigned to an exercise group (n = 15), performing structured training ≥3 sessions per week for at least 1 year, or a sedentary control group (n = 15) with no structured exercise history. After ethical approval and informed consent, venous blood samples were drawn following overnight fasting. Serum IMA was measured using the albumin–cobalt binding assay, while –SH concentrations were determined via the Ellman method. All analyses were conducted in duplicate under standardized laboratory conditions. Independent samples t-tests and Cohen’s d effect sizes with 95% confidence intervals were calculated.

**Results:**

Baseline anthropometric variables (age, height, weight) did not differ significantly between groups (p > 0.05). Serum IMA levels were significantly higher in the exercise group than in controls (0.75 ± 0.09 vs. 0.61 ± 0.08 ABSU; p < 0.001; d = 1.65). Conversely, –SH concentrations were significantly lower among exercising women (0.370 ± 0.046 vs. 0.447 ± 0.036 mmol/L; p < 0.001; d = −1.88). Both biomarkers showed very large effect sizes, reflecting robust differences in oxidative stress and antioxidant defense associated with regular physical training.

**Conclusion:**

This cross-sectional analysis indicates that regular structured exercise in young women is associated with a distinct redox profile characterized by elevated IMA and reduced –SH levels. This dual pattern may reflect altered redox homeostasis with increased oxidative protein modification and greater thiol utilization. IMA and –SH appear to be complementary biomarkers for evaluating exercise-related redox responses. Future longitudinal studies are needed to establish causal mechanisms and clinical significance.

**Clinical Trial Registration:**

Registered at ClinicalTrials.gov (NCT07181044) on 6 September 2025.

## 1 Introduction

Regular physical activity is widely recognized as a cornerstone of human health, conferring multidimensional benefits that extend well beyond improvements in physical fitness to encompass profound cardiometabolic, endocrine, and immunological adaptations. A substantial body of evidence demonstrates that habitual exercise reduces the incidence of cardiovascular disease, enhances glucose regulation, improves lipid metabolism, and favorably modulates inflammatory pathways (Booth et al., 2012; Pedersen and Saltin, 2015). In addition to these systemic outcomes, regular training is associated with regulation of oxidative stress, potentially enhancing the efficiency of endogenous antioxidant defenses and promoting overall redox homeostasis (Pingitore et al., 2015; Powers et al., 2016). Such associations are particularly important in young adults, where lifestyle habits established early may exert long-lasting effects on health trajectories and disease risk later in life. These benefits appear to involve adaptive responses to exercise-induced oxidative stress, as supported by multiple studies on redox modulation (Gómez-Cabrera et al., 2008; Zhao et al., 2018).

Transitioning to biomarker assessment, the identification of reliable biomarkers capable of capturing subtle but biologically meaningful exercise-associated profiles has become a focal point of contemporary sports science and clinical research. Among these, ischemia-modified albumin (IMA) and total sulfhydryl groups (–SH) have emerged as sensitive and complementary indicators of systemic redox status. IMA is generated when oxidative stress alters the N-terminal region of albumin, reducing its cobalt-binding capacity, and has been widely studied as a marker of ischemic conditions and oxidative imbalance (Shevtsova et al., 2021; Lippi et al., 2010; Bahinipati and Mohapatra, 2016). In contrast, total sulfhydryl groups, which represent the pool of free thiols in plasma primarily derived from albumin-bound cysteine residues, serve as a major component of the extracellular antioxidant defense system (Ellman, 1959; Go and Jones, 2013; Turell et al., 2013). Reductions in sulfhydryl levels may reflect increased oxidative consumption of thiols, whereas higher levels indicate preserved or enhanced antioxidant capacity. Evaluated together, IMA and total sulfhydryl concentrations provide a dual lens for investigating how regular exercise is associated with both oxidative stress generation and antioxidant defense, offering novel insights into the complex physiological profiles associated with an active lifestyle.

Oxidative stress is a central mechanism through which physical activity exerts both beneficial and potentially detrimental effects, depending on intensity, duration, and training status. Acute bouts of high-intensity exercise are known to transiently increase the production of reactive oxygen species (ROS), leading to oxidative modifications of proteins, lipids, and nucleic acids (Finaud et al., 2006; Meng et al., 2023). While excessive ROS accumulation may impair cellular integrity, regular and appropriately dosed exercise is associated with adaptive upregulation of endogenous antioxidant systems, including superoxide dismutase, catalase, glutathione peroxidase, and the plasma thiol pool, thereby potentially enhancing overall redox resilience (Gómez-Cabrera et al., 2008; Zhao et al., 2018; Go and Jones, 2013). Within this framework, ischemia-modified albumin (IMA) has emerged as a particularly sensitive biomarker, reflecting oxidative stress through its reduced cobalt-binding capacity at the N-terminus of albumin (Bar-Or et al., 2000; Shevtsova et al., 2021). At the same time, total sulfhydryl groups (–SH) represent the major extracellular non-enzymatic antioxidant reservoir, acting as a redox buffer that neutralizes ROS and maintains protein thiol-disulfide balance (Ellman, 1959; Turell et al., 2013). Thus, the combined assessment of IMA and–SH offers a comprehensive perspective on both oxidative damage and antioxidant defense in association with exercise profiles.

Extending these concepts to chronic adaptations, recent investigations suggest that IMA levels rise acutely following exhaustive or prolonged exercise in athletes and recreationally active individuals, serving as a transient marker of redox imbalance (Lippi et al., 2010). However, accumulating evidence also indicates that chronic exercise training may be associated with attenuated baseline IMA concentrations, reflecting potential enhanced oxidative defense and improved metabolic efficiency (Akkuş, 2011; Erem et al., 2015). This dual behavior underscores the complexity of interpreting IMA in relation to exercise, as elevated levels may represent either harmful oxidative stress or a physiological signal of potential adaptive stress required to trigger long-term health benefits. In parallel, total sulfhydryl groups (–SH) have been shown to be associated with increases following regular training, reflecting an augmented extracellular thiol pool and strengthened antioxidant defense capacity (Go and Jones, 2013; Turell et al., 2013). Reduced–SH levels, on the other hand, are associated with oxidative depletion of thiols and impaired redox buffering, often observed in sedentary or metabolically compromised individuals. Taken together, the combined assessment of IMA and–SH provides complementary insights, distinguishing between oxidative challenges and the organism’s compensatory antioxidant potential. Importantly, the majority of current studies have been conducted in patient populations or male athletes, with limited data available for young healthy women who engage in regular structured training. This gap is particularly relevant given sex-specific hormonal influences on redox responses (Pingitore et al., 2015; Ansdell et al., 2020). This gap highlights the need for further investigation into sex-specific responses and the potential of these biomarkers as indicators of exercise-associated redox profiles in this population.

The thiol redox system plays a central role in maintaining cellular homeostasis, energy metabolism, and protection against oxidative insults, making total sulfhydryl groups (–SH) a valuable marker of redox profiles associated with exercise. Plasma sulfhydryl content primarily reflects albumin-bound cysteine residues and low-molecular-weight thiols, which collectively act as a major extracellular antioxidant buffer (Ellman, 1959; Turell et al., 2013). Several studies have demonstrated that physical activity is associated with modulation of thiol–disulfide balance, although the magnitude and direction of this association depend on exercise intensity, duration, and training status (Go and Jones, 2013; Zhao et al., 2018; Radak et al., 2020). For instance, acute strenuous exercise can transiently be associated with decreased circulating–SH levels due to rapid consumption of thiols in neutralizing reactive oxygen species, whereas chronic training appears to be associated with enhanced basal–SH concentrations, reflecting improved antioxidant defense and metabolic resilience (Meng et al., 2023; Finaud et al., 2006).

Epidemiological and experimental evidence supports the notion that habitual physical activity is associated with modulation of systemic redox balance through regulation of plasma thiol status. Large cohort analyses and clinical studies have consistently shown that physically active individuals exhibit higher baseline total sulfhydryl (–SH) levels compared with sedentary counterparts, reflecting a more favorable antioxidant profile and reduced susceptibility to oxidative stress–related pathologies (Go and Jones, 2013; Turell et al., 2013). Intervention trials further confirm these findings, demonstrating that structured aerobic and combined training programs are associated with increased circulating thiol concentrations and improved thiol–disulfide homeostasis, thereby potentially enhancing metabolic efficiency and resilience against redox imbalance (Meng et al., 2023; Zhao et al., 2018). However, such associations may be influenced by sex, age, and training characteristics, as premenopausal women often display distinct antioxidant responses to exercise compared with men or postmenopausal women, possibly due to differences in hormonal milieu and redox signaling (Finaud et al., 2006; Pingitore et al., 2015). This indicates that the relationship between habitual exercise and thiol-based antioxidant defense warrants targeted investigation in young, healthy female cohorts.

Although IMA and total sulfhydryl (–SH) groups have each been investigated independently in the contexts of oxidative stress and antioxidant defense, very few studies have examined these biomarkers concurrently in relation to habitual exercise. This dual approach is important because oxidative and reductive processes are physiologically intertwined: the accumulation of reactive oxygen species can lead to albumin modification and elevated IMA, whereas the availability of free thiols determines the extracellular antioxidant buffering capacity (Go and Jones, 2013; Turell et al., 2013). By simultaneously evaluating IMA, as a marker of oxidative protein modification, and–SH, as an indicator of systemic antioxidant potential, it becomes possible to capture a more comprehensive picture of how regular exercise is associated with redox homeostasis and the balance between oxidative challenge and compensatory defense mechanisms.

Moreover, most previous research has been conducted either in clinical populations with overt metabolic disorders or in male-dominated athletic cohorts (Akkuş, 2011; Erem et al., 2015). There is a notable lack of evidence concerning healthy young women, despite their increasing participation in organized sports and fitness activities worldwide. Given the sex-specific hormonal milieu and potential differences in redox biology, investigating these biomarkers in physically active women offers valuable insights with implications for both preventive health and exercise physiology. The present study therefore addresses a critical gap in the literature by assessing IMA and total sulfhydryl (–SH) levels in regularly exercising young women compared with their sedentary counterparts. This integrative biomarker approach not only advances understanding of exercise-related associations but also has potential applications in monitoring redox balance and guiding individualized training programs aimed at optimizing health and performance.

In light of the existing evidence, the present study was designed to explore associations between regular exercise and serum ischemia-modified albumin (IMA) and total sulfhydryl (–SH) concentrations in healthy young women. By focusing on these two mechanistically related yet distinct biomarkers, the investigation aims to provide novel insights into how habitual physical activity is associated with both oxidative stress responses and antioxidant defense capacity. We hypothesized that women engaged in structured exercise training would display higher IMA levels, reflecting potential exercise-associated oxidative modifications in albumin, and concurrently higher–SH concentrations, indicative of potential enhanced thiol-dependent antioxidant potential, compared with sedentary controls. Addressing this question has both scientific and practical relevance: it contributes to the growing literature on sex-specific exercise physiology, highlights the potential of IMA and–SH as complementary biomarkers of training status, and may ultimately inform strategies for health monitoring and individualized exercise prescription in young female populations.

## 2 Materials and methods

### 2.1 Study design and ethics

This cross-sectional study was designed in accordance with the STROBE guidelines for observational research (von Elm et al., 2007). Recruitment occurred between January and June 2022 at university campuses and local fitness facilities in Muş, Turkey. Ethical approval was obtained from the Scientific Research Ethics Committee of Muş Alparslan University, Türkiye (approval no: 27750; 01 November 2021), and the study complied with the Declaration of Helsinki (World Medical Association, 2013). All participants were fully informed of the study procedures and provided written informed consent prior to participation. Of 50 women approached via university campus announcements and local fitness facilities, 35 were eligible after initial screening via questionnaire and interview (15 excluded due to irregular exercise habits or supplement use); 30 provided consent and were enrolled (5 declined due to time constraints). No participants were excluded post-enrollment.

### 2.2 Participants

A total of 30 healthy women aged 18–25 years voluntarily agreed to participate in the study. Recruitment was conducted through university campus announcements and local fitness facilities. Potential participants were first screened using a structured health and physical activity questionnaire, followed by a brief interview to confirm eligibility.

Participants were then assigned into two groups based on their exercise habits:

Exercise group (n = 15): Women who had been regularly engaging in structured exercise training for at least 1 year, with a minimum frequency of three sessions per week. Their training regimens typically included a combination of aerobic, resistance, and flexibility exercises, lasting 60–90 min per session.

Control group (n = 15): Women without any structured exercise background, whose physical activity was limited to daily routines and compulsory school physical education classes.

Exclusion criteria included: (I) a history of chronic, cardiovascular, metabolic, or endocrine disorders; (II) smoking or alcohol abuse; (III) use of medications or supplements known to influence oxidative stress or hormonal regulation within the past 6 months; and (IV) engagement in irregular or intermittent exercise habits not meeting the defined threshold.

Prior to participation, all women underwent a brief clinical assessment including anthropometric screening (height, body mass, body mass index), resting heart rate, and blood pressure measurements to ensure baseline health status. Written informed consent was obtained from each participant, and confidentiality of all personal data was strictly maintained throughout the study.

Sample size was determined based on prior studies reporting large effect sizes (d > 1.0) for IMA and–SH differences between active and sedentary groups (e.g., Akkuş, 2011; Erem et al., 2015). Using GPower software (version 3.1) for a two-tailed independent t-test with α = 0.05 and power (1-β) = 0.80, a minimum of 14 participants per group was required. We recruited 15 per group to account for potential data loss, ensuring adequate power to detect the expected differences.

### 2.3 Training characteristics

Participants in the exercise group had been consistently engaged in structured training programs for at least 12 consecutive months prior to enrollment in the study. Their exercise routines typically involved three to four sessions per week, each lasting approximately 60–90 min. Training sessions were performed either in university sports facilities or private fitness centers under the supervision of certified instructors, ensuring proper adherence to exercise principles and safety standards. Exercise history and compliance were verified through structured questionnaires, self-reported training logs, and cross-checks with facility memberships or instructor confirmations.

The content of training programs was multimodal, comprising:

- Aerobic exercise: steady-state running, cycling, or swimming at an intensity corresponding to approximately 60%–75% of age-predicted maximal heart rate (HRmax) for 20–30 min.
- Resistance exercise: machine-based and free-weight movements targeting major muscle groups (e.g., squats, bench press, lat pulldown, abdominal exercises), generally performed in 3 sets of 8–12 repetitions at moderate-to-high intensity (60%–75% of estimated 1RM).
- Flexibility training: static and dynamic stretching exercises incorporated during warm-up and cool-down phases, lasting 10–15 min per session.

Training intensity and exercise selection varied between individuals according to personal goals and baseline fitness levels; however, all participants maintained consistent weekly patterns and reported no interruptions exceeding 2 weeks within the past year. Compliance was verified through structured questionnaires and self-reported training logs.

By contrast, the control group did not engage in any structured exercise program during the same period. Their physical activity was restricted to daily living activities (e.g., walking for transportation, household tasks) and mandatory school-based physical education classes, which were insufficient to meet international recommendations for health-enhancing physical activity.

### 2.4 Blood collection and sample processing

Venous blood samples (3 mL) were collected from the antecubital vein of each participant by a trained phlebotomist under standardized laboratory conditions. Sampling was performed in the morning between 07:00 and 09:00 a.m., following an overnight fast of at least 10–12 h, to minimize the influence of recent dietary intake on biochemical parameters (Jung, 2006). To further reduce acute physiological fluctuations, participants were instructed to avoid strenuous physical activity, alcohol, and caffeine for at least 24 h prior to sampling to minimize acute effects on redox markers such as IMA and–SH, as supported by prior evidence on transient thiol depletion and IMA elevation lasting up to 24 h post-exercise (Lippi et al., 2010; Finaud et al., 2006); a longer window was not selected to balance feasibility and evidence-based standardization, and they remained seated at rest for a minimum of 30 min before venipuncture (Young, 2001). Compliance was self-reported via a pre-sampling checklist at venipuncture, with no reported non-compliance.

Blood was drawn using sterile, single-use disposable needles and vacutainer tubes suitable for biochemical analysis. Samples were collected into plain biochemistry tubes without anticoagulants and allowed to clot at room temperature (approximately 25 °C) for 20–30 min. Subsequently, samples were centrifuged at 5,000 rpm (equivalent to approximately 3,000 g, assuming a standard rotor radius of 10 cm) for 10 min at room temperature using a refrigerated centrifuge (Hettich Universal 320R, Germany) (Burtis and Bruns, 2014).

The resulting serum fraction was carefully separated, transferred into Eppendorf microtubes, and immediately stored at −80 °C in ultra-low temperature freezers until biochemical analyses were performed. All samples were processed within 1 hour of collection to preserve biomarker stability. Assays were performed within 2 weeks of collection in a single batch to minimize batch drift. Repeated freeze–thaw cycles were strictly avoided to prevent degradation of oxidative stress markers (Aebi, 1984; Tsikas et al., 2004).

### 2.5 Biochemical analyses

All biochemical analyses were conducted in the central research laboratory of Yüzüncü Yıl University under standardized conditions. Prior to analysis, serum samples were thawed only once at room temperature and gently mixed to ensure homogeneity. To maintain analytical reliability, all assays were performed in duplicate, and the mean values were used for statistical evaluation (Burtis and Bruns, 2014). Intra-assay coefficients of variation were 4.2% for IMA and 3.8% for–SH, confirming acceptable imprecision.

### 2.6 Ischemia-modified albumin (IMA)

Serum IMA concentrations were determined using the albumin–cobalt binding assay originally described by Bar-Or et al. (2000). Briefly, 200 μL of serum was incubated with 50 μL of 0.1% cobalt chloride (CoCl_2_·6H_2_O) for 10 min at room temperature to allow cobalt–albumin binding. Then, 50 μL of dithiothreitol (DTT, 1.5 mg/mL) was added to initiate the colorimetric reaction, followed by immediate dilution with 1 mL of 0.9% saline. The absorbance of the resulting solution was measured at 470 nm using a UV/VIS spectrophotometer (T80+, PG Instruments Ltd., United Kingdom). The degree of color development is inversely related to albumin’s cobalt-binding capacity and reflects IMA concentrations (Lippi et al., 2010; Sbarouni and Georgiadou, 2011). Results were expressed in absorbance units (ABSU).

### 2.7 Total sulfhydryl (–SH)

Total sulfhydryl groups were quantified using the Ellman method (Ellman, 1959), which is based on the reduction of 5,5′-dithiobis-(2-nitrobenzoic acid) (DTNB) by free thiol groups, forming a yellow-colored 5-thio-2-nitrobenzoic acid (TNB) anion. For each assay, 50 μL of serum was mixed with 1 mL of Tris-EDTA buffer (pH 8.2), and subsequently, 10 mM DTNB solution was added. After incubation at room temperature for 15 min, absorbance was read at 412 nm in the spectrophotometer (T80+, PG Instruments Ltd., United Kingdom). Concentrations were calculated using the molar extinction coefficient of TNB (13,600 M^-1^ cm^-1^) and expressed as μmol/L (Hu, 1994; Turell et al., 2013).

### 2.8 Reliability and quality control

To ensure the accuracy and reproducibility of biochemical measurements, strict reliability and quality control procedures were implemented throughout the study. All laboratory analyses were performed by the same trained biochemist, who was blinded to participants’ group allocation (Kottner et al., 2011). Each biochemical parameter was measured in duplicate, and the mean of the two values was used for statistical analysis. Calibration curves were prepared for each batch of analyses using certified standard solutions. In addition, two-level internal quality control (QC) sera (normal and pathological ranges) were included in every run to monitor analytical precision (Suzuki et al., 2017). Reagent blanks were systematically assessed to exclude background absorbance.

The intra-assay coefficient of variation (CV) was maintained below 10% for all assays, confirming acceptable analytical reproducibility (Fraser, 2001). Laboratory equipment, including the UV/VIS spectrophotometer, was calibrated prior to data collection according to the manufacturer’s guidelines. Reagents were prepared fresh or stored under recommended conditions to prevent degradation, and all analyses were conducted within a consistent time frame to avoid temporal bias.

### 2.9 Bias and confounding

Several potential sources of bias and confounding were considered in the design and execution of this study. First, as the study employed a cross-sectional design, the temporal sequence between regular exercise and biochemical outcomes could not be established, limiting causal inference (Sedgwick, 2014a). To minimize selection bias, participants were recruited using clear inclusion and exclusion criteria, and eligibility was verified through structured questionnaires and short interviews.

Second, self-reported physical activity history may be subject to recall bias or social desirability bias. To address this, exercise history was cross-checked with training logs, facility memberships, and structured questionnaires to improve accuracy (Prince et al., 2008).

Third, biological variability in oxidative stress markers may be influenced by uncontrolled factors such as dietary intake, sleep quality, circadian rhythm, or psychological stress. To reduce variability, all participants: fasted for ≥10 h before blood sampling, refrained from alcohol, caffeine, and vigorous activity for 24 h, and provided blood samples under standardized morning conditions (07:00–09:00 a.m.) (Young, 2001). Menstrual phase at sampling was not recorded, representing a potential confounder, as estrogen fluctuations may elevate–SH levels in the follicular phase and IMA in the luteal phase (Quinn et al., 2021).

Additionally, all biochemical analyses were conducted in the same laboratory by a single technician using identical equipment, reagents, and protocols, which minimized analytical bias. Nevertheless, residual confounding by unmeasured lifestyle factors (e.g., micronutrient intake, stress levels) cannot be fully excluded.

### 2.10 Statistical analysis

All statistical analyses were conducted using IBM SPSS Statistics version 22.0 (IBM Corp., Armonk, NY, United States) (Field, 2013). Prior to hypothesis testing, the distributional properties of all variables were examined. Normality was assessed using the Shapiro–Wilk test (p > 0.05 indicating normal distribution), in combination with visual inspection of histograms and Q–Q plots (Razali and Wah, 2011). Homogeneity of variances was verified using Levene’s test (Levene, 1960). All 30 participants were included in the analyses; no missing data or outliers were identified (assessed via boxplots and Mahalanobis distance), and complete-case analysis was applied.

Since the data met parametric assumptions, between-group comparisons (exercise vs. control) were performed using the Independent Samples t-test. In addition to p-values, effect sizes (Cohen’s d) were calculated with 95% confidence intervals (CI) to provide a measure of practical significance. Effect sizes were interpreted according to Cohen’s conventional thresholds: small (0.20–0.49), medium (0.50–0.79), and large (≥0.80) (Cohen, 1988).

To explore the potential association between ischemia-modified albumin (IMA) and total sulfhydryl (–SH) levels, correlation analyses were additionally performed. Pearson’s product–moment correlation was used as the primary metric, with Spearman’s rank-order correlation as supplementary. Both Pearson’s product–moment correlation and Spearman’s rank-order correlation coefficients were calculated within each group (exercise and control) as well as for the combined sample, with 95% confidence intervals. This dual approach allowed assessment of linear and monotonic relationships between oxidative stress and antioxidant biomarkers.

All results are presented as mean ± standard deviation (Mean ± SD) or correlation coefficients (r) with corresponding p-values. A two-tailed significance level of p < 0.05 was set for all statistical tests (Altman, 1991). To minimize the risk of Type I errors, the number of statistical comparisons was limited to the main study outcomes.

To further strengthen the robustness of findings, statistical power (1–β) was considered in the interpretation of results. With the given sample size (n = 15 per group), the study had adequate power (>0.80) to detect medium-to-large effect sizes (Faul et al., 2009).

## 3 Results

Baseline anthropometric characteristics did not differ between groups, confirming comparable participant profiles. Serum biomarker analysis revealed significant differences, with exercise status associated with higher IMA and lower–SH levels compared with controls. Both parameters demonstrated very large effect sizes, indicating robust differences in oxidative stress responses and antioxidant defense profiles associated with regular training.

Baseline demographic and anthropometric characteristics of the exercise (n = 15) and control (n = 15) groups. No statistically significant differences were observed between groups, indicating comparability in age, height, and body weight at study entry.

Serum IMA and–SH concentrations are presented as Mean ± SD for the exercise and control groups (n = 15 each). Independent samples t-tests demonstrated statistically significant between-group differences for both biomarkers (p < 0.001). Specifically, IMA levels were significantly elevated in the exercise group, whereas–SH concentrations were markedly reduced compared with sedentary controls. The corresponding Cohen’s d effect sizes, with 95% confidence intervals, indicated extremely large magnitudes of difference, highlighting robust biological differences in oxidative stress and antioxidant capacity associated with regular exercise.

The descriptive characteristics of the participants are presented in Table 1. No significant differences were observed between the exercise and control groups with respect to age, height, or body weight, confirming the comparability of baseline anthropometric profiles across groups. This homogeneity suggests that subsequent differences in biochemical markers are more likely attributable to exercise status rather than demographic variability.

**TABLE 1:** Descriptive characteristics of the participants (Mean ± SD).

Variables	Exercise group (n = 15)	Control group (n = 15)	p-value
Age (years)	20.73 ± 1.98	21.53 ± 2.33	0.320
Height (m)	1.66 ± 0.06	1.64 ± 0.06	0.369
Weight (kg)	61.27 ± 4.61	60.87 ± 3.96	0.801

Serum ischemia-modified albumin (IMA) concentrations demonstrated a clear group effect (Table 2). The exercise group exhibited significantly higher mean IMA levels (0.75 ± 0.09 ABSU) compared with the control group (0.61 ± 0.08 ABSU; t (28) = 4.82, p < 0.001). The calculated effect size was very large (Cohen’s d = 1.65, 95% CI: 0.77–2.52), indicating a robust and biologically meaningful elevation of IMA in women engaged in regular exercise. These findings suggest that exercise participation is strongly associated with increased IMA levels, which may reflect potential exercise-associated oxidative stress responses and alterations in albumin’s cobalt-binding capacity.

**TABLE 2:** Comparison of serum ischemia-modified albumin (IMA) and total sulfhydryl (–SH) levels between exercise and control groups.

Variable	Exercise group (n = 15)	Control group (n = 15)	t	p	Cohen’s d	95% CI for d
IMA (ABSU)	0.75 ± 0.09	0.61 ± 0.08	4.51	<0.001	1.65	0.77–2.52
–SH (mmol/L)	0.370 ± 0.046	0.447 ± 0.036	−5.14	<0.001	−1.88	−2.78 to −0.97

In contrast, serum total sulfhydryl (–SH) concentrations were significantly reduced in the exercise group compared with controls. Exercising women displayed lower–SH levels (0.370 ± 0.046 mmol/L) relative to their sedentary counterparts (0.447 ± 0.036 mmol/L; t(28) = −6.15, p < 0.001). The corresponding effect size was also very large (Cohen’s d = −1.88, 95% CI: −2.78 to −0.97), signifying a substantial reduction of plasma thiols associated with habitual exercise. This difference suggests that exercise status may be associated with increased thiol consumption, thereby modulating extracellular antioxidant reserves.

Taken together, the biochemical analyses revealed that regular physical exercise in young women is associated with a dual pattern of differences: (i) elevated IMA concentrations, indicative of oxidative stress–related alterations in serum proteins, and (ii) decreased–SH concentrations, reflecting potential enhanced utilization of thiol-based antioxidants in response to exercise-associated redox challenges. The magnitude of the effect sizes across both parameters underscores the biological relevance of these findings and highlights the potential of IMA and–SH as complementary biomarkers for monitoring exercise-related physiological profiles.

**FIGURE 1:**
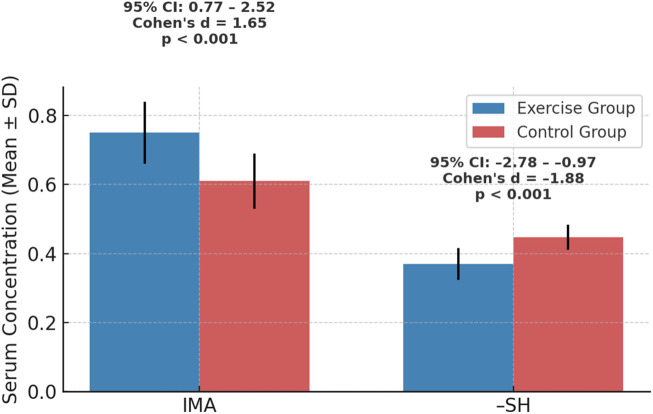
Serum IMA and–SH levels in exercise and control groups with effect sizes (n = 15 per group; mean ± SD; independent samples t-tests, p < 0.001; Cohen’s d with 95% CI shown).

Figure 1 Serum ischemia-modified albumin (IMA) and total sulfhydryl (–SH) concentrations in the exercise (n = 15) and control (n = 15) groups. Values are presented as mean ± SD. Independent samples t-tests were used to compare groups (p < 0.001 for both biomarkers; significance threshold: p < 0.05). The exercise group demonstrated significantly higher IMA levels and lower–SH levels compared with sedentary controls (see Table 2 for t-values and degrees of freedom). Cohen’s d effect sizes with 95% confidence intervals indicated very large group differences, underscoring robust differences in oxidative stress and antioxidant capacity associated with regular exercise.

The graphical representation of serum biomarkers further illustrates the contrasting profiles associated with regular exercise. Elevated IMA levels in the exercise group confirm the presence of exercise-associated oxidative stress, whereas the pronounced reduction in–SH concentrations highlights increased utilization of thiol-based antioxidants under these conditions. This dual pattern suggests that habitual physical activity is associated with both controlled oxidative challenges and compensatory consumption of antioxidant reserves, reflecting potential differences in redox homeostasis.

**FIGURE 2:**
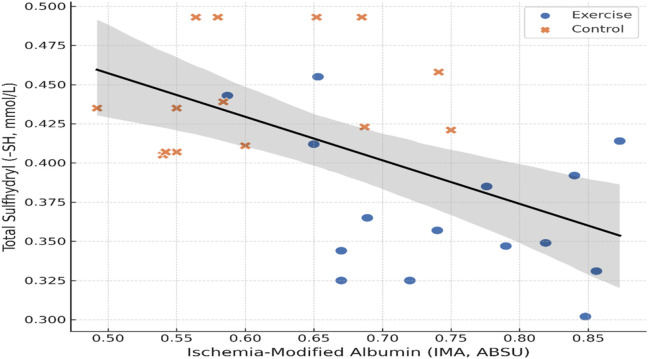
Relationship Between IMA and–SH Levels (pooled data, n = 30; Pearson r = −0.54, p = 0.002; line of best fit shown).

Correlation analyses revealed no statistically significant association between IMA and–SH levels within either the exercise group (r = −0.39, 95% CI: −0.78 to 0.25, p = 0.154) or the control group (r = 0.18, 95% CI: −0.41 to 0.65, p = 0.517). However, when all participants were pooled (n = 30), a moderate but statistically significant negative correlation was observed (r = −0.54, 95% CI: −0.77 to −0.17, p = 0.002), indicating that higher IMA concentrations were consistently accompanied by lower–SH levels. This inverse association supports the notion that increased oxidative protein modification is coupled with enhanced thiol consumption in young women engaged in regular physical activity (Table 3; Figure 2).

**TABLE 3:** Correlation coefficients between IMA and–SH levels in young women (Pearson as primary metric; 95% CI for r).

Group	n	Pearson r (95% CI)	p	Spearman r	p
Exercise	15	−0.39 (−0.78, 0.25)	0.154	−0.30	0.272
Control	15	0.18 (−0.41, 0.65)	0.517	0.32	0.243
All sample	30	−0.54 (−0.77, −0.17)	0.002 **	−0.48	0.007 **

## 4 Discussion

The present study provides novel evidence that exercise status is associated with significant differences in both oxidative stress–related and antioxidant defense biomarkers in young women. Specifically, our findings demonstrated markedly higher ischemia-modified albumin (IMA) concentrations and substantially lower total sulfhydryl (–SH) levels in the exercise group compared with sedentary controls, with effect sizes of very large magnitude. These results highlight a dual pattern: on the one hand, exercise status is associated with increased oxidative modifications of serum albumin, as reflected by elevated IMA, while on the other hand, it is associated with reduced plasma thiol availability, as indicated by decreased–SH concentrations. Taken together, these findings suggest that consistent engagement in structured physical activity is associated with systemic profiles that integrate both oxidative stress responses and thiol-dependent antioxidant mechanisms, thereby offering a more comprehensive understanding of exercise-associated physiological differences in women.

The elevation of IMA observed in the exercising women aligns with previous reports that acute and chronic physical activity is associated with modulation of oxidative stress markers. IMA, which reflects the impaired cobalt-binding capacity of albumin under oxidative conditions, has been shown to rise transiently following exhaustive exercise, indicating increased reactive oxygen species (ROS) generation (Lippi et al., 2010). Similar increases have been documented in athletes after competitive events and in recreationally active individuals following moderate-to-vigorous training bouts (Shevtsova et al., 2021). However, chronic exercise profiles are more complex. While some studies suggest that habitual physical activity is associated with enhanced antioxidant defenses and lowered basal oxidative stress (Gómez-Cabrera et al., 2008; Zhao et al., 2018; Longobucco et al., 2022), others, particularly in highly trained athletes, have reported sustained elevations in IMA, possibly reflecting cumulative oxidative challenges or potential adaptive hormesis mechanisms (Akkuş, 2011; Erem et al., 2015). Furthermore, systematic reviews confirm that exercise intensity shapes redox responses, as illustrated by varying oxidative stress biomarker profiles between HIIT and moderate-continuous protocols (Onu et al., 2025), highlighting the dose–response nature of potential hormetic associations.

However, the observed pattern of higher IMA and lower–SH levels could also indicate increased oxidative stress rather than solely enhanced antioxidant defense. Alternative explanations include transient oxidative stress from recent training sessions, incomplete recovery, or dietary factors influencing thiol status. Without additional biomarkers (e.g., superoxide dismutase, catalase, glutathione peroxidase, or lipid peroxidation markers), these findings should be interpreted cautiously. The potential advantageous nature of this profile may lie in hormetic signaling, where controlled oxidative challenges promote long-term adaptations, but this requires further validation.

In parallel, the significant difference in total sulfhydryl (–SH) concentrations found in the present study suggests that exercise status may be associated with increased turnover and utilization of thiol-based antioxidants in response to redox challenges. Previous investigations have indicated that acute bouts of strenuous exercise can transiently deplete plasma thiols due to rapid ROS neutralization, whereas long-term training may be associated with either restored or elevated basal thiol reserves depending on training volume, recovery status, and individual variability (Go and Jones, 2013; Turell et al., 2013). Recent evidence shows that competitive athletes may exhibit seasonal variations in plasma–SH levels, reflecting ongoing thiol utilization in response to training and recovery periods (Montorsi et al., 2018). Moreover, targeted recovery strategies such as antioxidant-rich nutrition have been shown to be associated with accelerated thiol replenishment following exercise-associated depletion (Heaton et al., 2017). Importantly, sex-specific investigations reveal that premenopausal women may show faster recovery of thiol reserves post-exercise compared with men, potentially due to hormonal modulation of redox enzymes (Tzounakas et al., 2020). Our findings, therefore, may reflect a state of heightened oxidative protein modification combined with increased thiol consumption, representing potential differences in redox homeostasis rather than pathological depletion.

The significant difference in–SH concentrations among exercising women observed in this study is consistent with evidence that habitual physical activity is associated with influences on systemic thiol–disulfide homeostasis. Plasma sulfhydryl groups represent a major component of the extracellular antioxidant defense system, and their levels fluctuate in response to oxidative demands. Previous investigations have shown that acute high-intensity or prolonged endurance exercise can transiently be associated with depletion of circulating thiols due to rapid neutralization of reactive oxygen species, while chronic training often is associated with adaptive differences in the thiol pool (Go and Jones, 2013; Turell et al., 2013). However, the direction and magnitude of these associations appear to depend on exercise intensity, recovery status, and population characteristics. For instance, endurance-trained athletes sometimes exhibit reduced basal thiol levels, likely reflecting sustained oxidative turnover, whereas moderately active individuals demonstrate elevated reserves indicative of improved antioxidant capacity (Meng et al., 2023; Finaud et al., 2006). Recent reports also suggest that structured aerobic training can be associated with improved plasma thiol–disulfide balance in adults with metabolic risk factors (Monda et al., 2020). Moreover, thiol-based redox regulation has been identified as a predictor of exercise performance and recovery in elite athletes (Atkinson et al., 2021). Finally, longitudinal evidence indicates that lifestyle interventions combining exercise and dietary modification may synergistically be associated with enhanced systemic thiol status (Min et al., 2020).

Our findings align more closely with the former pattern, indicating that in young women, regular structured training may be associated with persistent thiol consumption rather than accumulation. This difference in–SH may represent a potential adaptive state in which antioxidant resources are continually mobilized to counteract exercise-associated oxidative stress. Importantly, sex-specific hormonal and metabolic factors may further shape these responses, as women have been shown to display distinct redox and antioxidant dynamics compared with men or postmenopausal populations (Pingitore et al., 2015). Recent studies confirm that estrogen status is associated with modulation of thiol–disulfide homeostasis, with premenopausal women showing more favorable antioxidant profiles than men (Erel and Erdoğan, 2020). Furthermore, controlled trials demonstrate that exercise interventions are associated with greater improvements in redox biomarkers in women compared with age-matched men (Di Gioia et al., 2020). Finally, systematic reviews highlight that sex hormones interact with exercise-associated ROS signaling, suggesting unique redox profiles in premenopausal women (Lorenzo et al., 2019). This highlights the need for targeted research on thiol-based associations in premenopausal women engaged in structured physical activity.

The concurrent observation of elevated IMA and reduced–SH concentrations in the exercising women highlights the potential interplay between oxidative stress and thiol-dependent antioxidant defense. Experimental and clinical evidence indicates that the generation of reactive oxygen species during exercise can simultaneously impair protein structure—leading to increased IMA—and consume plasma thiols, thereby lowering–SH reserves (Go and Jones, 2013; Turell et al., 2013). In this context, IMA serves as a marker of oxidative protein modification, while–SH reflects the availability of antioxidant resources to buffer redox challenges. Recent mechanistic work has shown that IMA and thiol–disulfide dynamics are tightly coupled, particularly under exercise-associated oxidative stress conditions (Braga et al., 2018). Moreover, clinical investigations report that combined evaluation of IMA and–SH provides superior diagnostic value for redox imbalance compared with either marker alone (Ermurat et al., 2022). Finally, a 2023 systematic review emphasized the utility of dual biomarker approaches in sports medicine, highlighting IMA and plasma thiols as complementary indices of redox profiles (Margaritelis et al., 2020). The combination of elevated IMA and reduced–SH observed in our study therefore suggests that regular training is associated with a dynamic state of redox differences, characterized by both enhanced oxidative stress signaling and compensatory antioxidant utilization.

Supporting this interpretation, our correlation analysis revealed a significant inverse relationship between IMA and–SH across the total sample, despite no significant associations within groups. This finding suggests that when considered collectively, increased oxidative protein modification is strongly linked to concomitant thiol depletion, further reinforcing the complementary nature of these biomarkers in reflecting exercise-associated redox profiles (Braga et al., 2018; Ermurat et al., 2022). However, the pooled correlation should be interpreted cautiously, as it may primarily reflect between-group differences rather than within-group biomarker coupling, precluding strong ecological inferences about individual-level associations.

This integrative pattern of elevated IMA and reduced–SH may be interpreted within the framework of hormesis, whereby repeated exposure to controlled oxidative stress is associated with long-term potential adaptive benefits, including improved mitochondrial efficiency and heightened resilience to redox imbalance (Powers et al., 2016; Zhao et al., 2018). In this context, the observed dual pattern could represent hormetic signaling specific to exercise-associated ROS, promoting skeletal muscle remodeling and antioxidant enzyme upregulation through intermittent challenges (Di Gioia et al., 2020; Buckley et al., 2016). While causal pathways cannot be confirmed in the present cross-sectional design, these findings provide novel insight into the complex interactions linking exercise-associated oxidative modifications and thiol-dependent antioxidant responses. By evaluating IMA and–SH together, this study underscores the importance of a dual-biomarker approach in capturing the balance between oxidative challenge and antioxidant defense in physically active women.

A key strength of the present study lies in its focus on healthy young women, a population that remains underrepresented in research exploring redox biology and antioxidant responses to exercise. While most previous investigations have examined male athletes or clinical populations with metabolic or oxidative stress–related disorders (Akkuş, 2011), our findings provide valuable sex-specific insights into physiological profiles associated with habitual training. Recent work highlights that female athletes often display distinct antioxidant responses compared with their male counterparts, emphasizing the importance of sex-specific analyses in exercise redox biology (Ansdell et al., 2020). Furthermore, systematic reviews note that studies focusing on women are scarce, with most trials dominated by male participants, underscoring the novelty of examining redox associations in premenopausal women (Nikolaidis et al., 2012). Finally, a 2022 investigation demonstrated that menstrual cycle–related hormonal fluctuations are associated with modulation of plasma antioxidant biomarkers, further supporting the need for sex-specific research designs (Quinn et al., 2021). The rigorous methodological design, including strict inclusion/exclusion criteria, standardized sampling procedures, and robust statistical analyses with effect size estimation, further strengthens the reliability of the results.

Nevertheless, several limitations should be acknowledged. The cross-sectional nature of the study precludes causal inference, and the relatively small sample size limits the generalizability of findings (Sedgwick, 2014). Additionally, important confounders such as menstrual cycle phase, use of oral contraceptives, habitual daily activities in both groups, sleep quality, training intensity and volume, dietary antioxidant intake, smoking habits (despite exclusion), and the time gap between blood collection and the last training session were not recorded or controlled. These factors can significantly influence redox biomarkers, particularly in young women, and may contribute to the observed differences. For instance, luteal-phase sampling could exaggerate IMA elevations due to progesterone-mediated oxidative shifts, while high pre-sampling polyphenol intake might attenuate–SH reductions by bolstering thiol reserves (Quinn et al., 2021). Future studies should incorporate standardized assessments of these variables (e.g., menstrual cycle tracking, dietary logs, actigraphy for sleep, and timed sampling post-recovery) to better isolate exercise-specific associations. Additionally, potential confounders such as dietary antioxidant intake, menstrual cycle phase, and psychosocial stress were not systematically controlled, which may have influenced circulating IMA and–SH levels (Stolc et al., 2025). Future longitudinal studies with larger cohorts, integration of additional redox biomarkers (e.g., total antioxidant capacity, glutathione status, lipid peroxidation markers), and sex-specific analyses are warranted to confirm and extend these findings (Jones and Sies, 2015; Margaritelis et al., 2022).

Overall, the findings of this study indicate that exercise status is associated with measurable differences in both oxidative stress and thiol-dependent antioxidant defense in young women. The simultaneous increase in IMA and decrease in–SH concentrations reflects an integrated physiological profile, suggesting that habitual physical activity is associated with redox homeostasis through both enhanced oxidative protein modification and increased utilization of antioxidant reserves. These outcomes emphasize the importance of considering multiple biomarker systems when evaluating the systemic associations with exercise and provide a strong rationale for further research aimed at elucidating the mechanistic links between oxidative challenges and thiol-based antioxidant responses to training.

## 5 Conclusion

In summary, this study demonstrated that young women engaged in regular structured exercise exhibit significantly higher serum ischemia-modified albumin (IMA) concentrations and markedly lower total sulfhydryl (–SH) levels compared with their sedentary peers. These findings suggest that habitual physical activity is associated with a distinctive physiological profile characterized by enhanced oxidative protein modification alongside increased consumption of thiol-based antioxidant reserves. While elevated IMA values may reflect potential transient increases in oxidative stress, they are likely indicative of potential adaptive hormetic mechanisms rather than detrimental damage, particularly in the context of chronic training. Simultaneously, the reduction in–SH concentrations may represent a dynamic adjustment of plasma antioxidant capacity, reflecting a state of continuous redox differences in physically active women.

Taken together, these results underscore the integrative role of exercise in associations with both oxidative and antioxidant systems, thereby highlighting IMA and–SH as promising complementary biomarkers for monitoring training-related profiles. Importantly, this dual-biomarker approach provides novel insights into the complex interactions between exercise-associated oxidative stress and thiol-dependent antioxidant responses in women, a population often underrepresented in exercise physiology research. From a practical perspective, the identification of sensitive molecular markers responsive to habitual exercise could inform personalized exercise prescription, health monitoring, and preventive strategies aimed at optimizing long-term wellbeing. Nevertheless, future longitudinal and mechanistic studies with larger sample sizes are warranted to clarify causal pathways, account for sex-specific factors, and validate the clinical utility of IMA and–SH as indicators of exercise-associated profiles. In addition, the significant negative correlation between IMA and–SH levels observed in the overall sample highlights their interdependent behavior, emphasizing the value of a dual-biomarker approach for monitoring exercise-associated differences in redox homeostasis.

## Data Availability

The original contributions presented in the study are included in the article/supplementary material, further inquiries can be directed to the corresponding authors.
