# Supplementary material for: Effects of regular exercise on ischemia-modified albumin and total sulfhydryl levels in young women: a cross-sectional study
Source: Front Physiol. 2025 Nov 20;16:1719454. doi: 10.3389/fphys.2025.1719454 (PMC12675226; doi:10.3389/fphys.2025.1719454)

Supplementary Material

**Table 1.** Descriptive characteristics of the participants (Mean ± SD).

| Variables | Exercise Group (n = 15) | Control Group (n = 15) | p-value |
| --- | --- | --- | --- |
| Age (years) | 20.73 ± 1.98 | 21.53 ± 2.33 | 0.320 |
| Height (m) | 1.66 ± 0.06 | 1.64 ± 0.06 | 0.369 |
| Weight (kg) | 61.27 ± 4.61 | 60.87 ± 3.96 | 0.801 |

**Table 2.** Comparison of serum ischemia-modified albumin (IMA) and total sulfhydryl (–SH) levels between exercise and control groups.

| Variable | Exercise Group (n=15) | Control Group (n=15) | t | p | Cohen’s d | 95% CI for d |
| --- | --- | --- | --- | --- | --- | --- |
| IMA (ABSU) | 0.75 ± 0.09 | 0.61 ± 0.08 | 4.51 | <0.001 | 1.65 | 0.77 – 2.52 |
| –SH (mmol/L) | 0.370 ± 0.046 | 0.447 ± 0.036 | –5.14 | <0.001 | –1.88 | –2.78 – –0.97 |

**Fig. 1** Serum IMA and –SH levels in exercise and control groups with effect sizes (n=15 per group; mean ± SD; independent samples t-tests, p < 0.001; Cohen’s d with 95% CI shown).


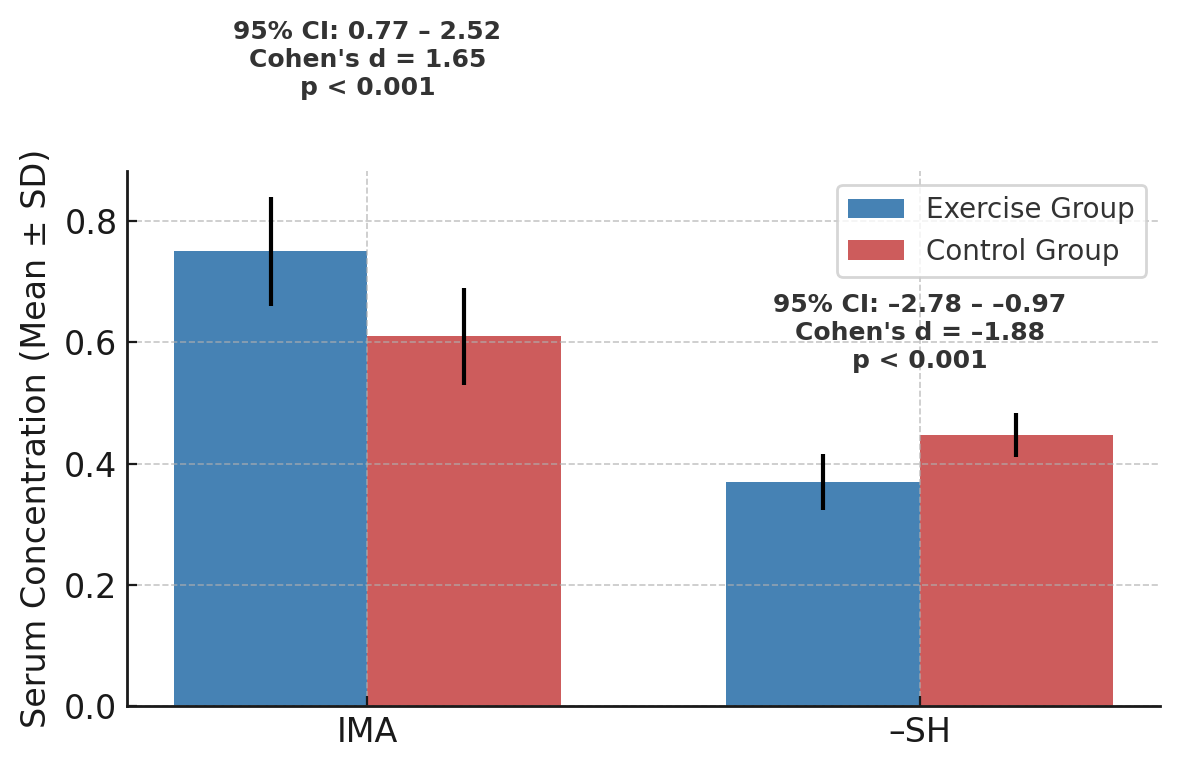


Table 3. Correlation coefficients between IMA and –SH levels in young women (Pearson as primary metric; 95% CI for r).

| Group | n | *Pearson r (95% CI)* | p | Spearman r | p |
| --- | --- | --- | --- | --- | --- |
| Exercise | 15 | –0.39 *(-0.78, 0.25)* | 0.154 | –0.30 | 0.272 |
| Control | 15 | 0.18 *(-0.41, 0.65)* | 0.517 | 0.32 | 0.243 |
| All sample | 30 | –0.54 *(-0.77, -0.17)* | 0.002 ** | –0.48 | 0.007 ** |

Fig. 2. Relationship Between IMA and –SH Levels (pooled data, n=30; Pearson r = –0.54, p=0.002; line of best fit shown).


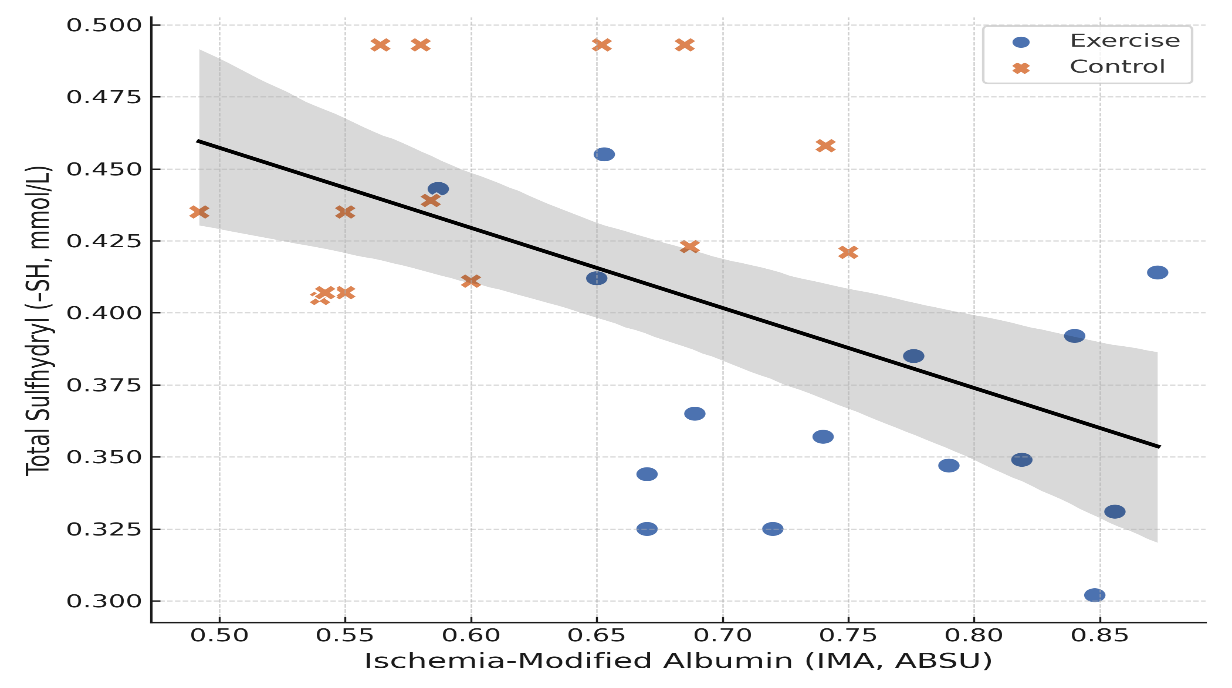

Supplement: Supplementary file 1 [file Supplementaryfile1.docx]
